# Reflections on the 2021 World Malaria Report and the future of malaria control

**DOI:** 10.1186/s12936-022-04178-7

**Published:** 2022-05-27

**Authors:** April Monroe, Nana Aba Williams, Sheila Ogoma, Corine Karema, Fredros Okumu

**Affiliations:** 1 Johns Hopkins Center for Communication Programs, Baltimore, USA; 2 MESA Alliance, Barcelona Institute for Global Health (ISGlobal), Barcelona, Spain; 3 Barcelona Institute for Global Health (ISGlobal), Hospital Clínic-Universitat de Barcelona, Barcelona, Spain; 4 Clinton Health Access Initiative, Boston, USA; 5 Quality and Equity Healthcare, Kigali, Rwanda; 6 Swiss Tropical & Public Health Institute, Basel, Switzerland; 7 Ifakara Health Institute, Ifakara, Tanzania

**Keywords:** World Malaria Report, Biological threats, COVID19

## Abstract

The World Malaria Report, released in December 2021, reflects the unique challenges currently facing the global malaria community. The report showed the devastating toll of malaria, with an estimated 627,000 people losing their lives to the disease in 2020. The improved methodological approach used for calculating cause of death for young children revealed a systematic underestimation of disease burden over the past two decades; and that Africa has an even greater malaria crisis than previously known. While countries were able to prevent the worst-case scenarios, the disruptions due to the COVID-19 pandemic revealed how weak health systems and inadequate financing can limit the capacity of the continent to address the malaria challenge. African countries also face a convergence of biological threats that could redefine malaria control, notably widespread pyrethroid resistance and emerging resistance to artemisinin. Despite these challenges, there is cause for optimism in lessons learned from the COVID-19 pandemic, recent acceleration of cutting edge research and development, and new partnerships that encourage leadership from and ownership by affected countries. This article presents key insights from the 2021 World Malaria Report and reflections on the future trajectories: it was informed by an in-depth discussion with leading malaria experts from the World Health Organization (WHO), the Bill & Melinda Gates Foundation, and the U.S. President’s Malaria Initiative (PMI). The discussion took place during the 34th edition of the Ifakara Master Classes, held virtually on December 15th, 2021.

## Background

On December 15th, 2021, the 34th edition of the Ifakara Master Classes featured an in-depth discussion on the 2021 World Malaria Report (WMR), released a week earlier [1]. The discussion unpacked WMR findings and their implications for the future of malaria control. Guest experts included Dr. Pedro Alonso, Director of the Global Malaria Programme (GMP) at the World Health Organization (WHO), Dr. Abdisalan Noor, WHO Head of Strategic Information for Response Unit, Dr. Jennifer Gardy, Deputy Director, Surveillance, Data, and Epidemiology at the Bill & Melinda Gates Foundation, and Dr. Richard Steketee, Deputy Global Malaria Coordinator for the U.S. President’s Malaria Initiative (PMI).

The discussion, which lasted 2 h and 45 min in total, was organized and facilitated by MasterClass hosts Drs. Fredros Okumu (Director of Science, Ifakara Health Institute, Tanzania) and Sheila Ogoma (Technical Director, Clinton Health Access Initiative), and guest hosts, Drs. Corine Karema (Private Consultant and former Director of National Malaria Control Programme, Rwanda) and Nana Aba Williams (Coordinator, MESA Alliance, ISGlobal, Spain). The session began with a brief overview of the 2021 WMR by Dr. Noor, followed by a series of open-ended technical questions posed by the facilitators to the panel of experts about specific aspects of the WMR. The discussion was hosted on Zoom with 320 live participants from the global malaria community, and was live-streamed on YouTube.

A consolidated account of insights and lessons learned from the discussion is presented here. Findings are organized around topics identified a priori by the Master Class facilitators and key themes that emerged through the discussion.

## The importance of numbers

The World Malaria Report, released December 2021, reflects the unique challenges facing the global malaria community. The report lays bare the devastating toll of malaria, with an estimated 627,000 people losing their lives to the disease in 2020. The numbers in the report tell two different stories for countries nearing elimination and countries experiencing high burden.

“A growing number of countries with low burden are moving steadily toward elimination, while countries with the highest burden are struggling.” –Dr. Noor

Eleven countries now experience 70% of the world’s malaria burden while 47 now report fewer than 10,000 cases per year. Even before the COVID-19 pandemic, gains against malaria were leveling off, leading to the role out of the High burden, High impact response in 2018 [2].

### Methodological changes

A new statistical method is being used by the WHO, which provides more precise cause-of-death estimates for young children for all diseases, including malaria. In the revised approach, the proportion of childhood deaths attributable to malaria was 7.8%, up from previous estimates of 4.8% [3, 4]. The revised approach revealed that there had been a higher number of estimated deaths between 2000 and 2020 than previously recognized and a systematic underestimation across the time series. The revisions also suggest that a higher number of malaria cases (totaling 1.7 billion) and deaths (10.6 million) had been averted in the same period.

The WMR has gotten clearer, and the quality improved consistently since it was first released. However, for most countries, the WHO still relies on modelled estimates derived from verbal autopsies to calculate all-cause mortality and the cause of death fraction for children under-5 to quantify malaria deaths in this age-group before applying a second adjustment to quantify deaths in older children and adults. There is a strong case for improving surveillance as an intervention and investing more heavily in information systems as recommended in the WHO Global Technical Strategy (GTS) 2016–2030 [5]. These malaria metrics, whether estimates or not, can be powerful advocacy tools and are, therefore, integral for creating compelling narratives of changes over time.

### Impact of COVID-19

In addition to increases due to the methodological changes, the COVID-19 pandemic posed significant challenges, and was associated with ~ 47,000 of the ~ 69,000 extra deaths reported in 2020 relative to 2019, [1]. This includes increases in cases due to disruptions associated with delays in ITN distribution and disruptions in both diagnosis and treatment. Malaria deaths increased by 12% to an estimated 627,000 in 2020, compared to 2019 figures, with more than two-thirds of the additional 69,000 deaths attributable to COVID19-related service disruptions. While the figures are worrying, countries and partners have done well to prevent the worst-case scenarios earlier projected by the WHO and partners [6–8]; these models had predicted increases in malaria cases and deaths in Africa of as much as two orders of magnitude.

## Threats to malaria control in Africa

A range of challenges from biological threats, to preventing severe disease and death in the most remote areas, to fragile and insufficient malaria funding must be addressed to sustain progress.

*“*The situation remains precarious, particularly in sub-Saharan Africa where burden remains unacceptably high and a convergence of threats pose added challenges to disease control efforts…Without immediate accelerated action, key 2030 targets of the WHO Global Technical Strategy [5] for malaria will be missed, and additional ground may be lost.” –Dr. Noor

### Biological threats

While the epidemiology of malaria in Africa is already more challenging and precarious than elsewhere, the situation is compounded by multiple biological and civil threats. Over 122 million people in 21 malaria-endemic countries needed assistance due to health and humanitarian emergencies in 2020–2021 including Ebola outbreaks, armed conflicts, and flooding. Key biological threats in sub-Saharan Africa include anti-malarial drug resistance in the eastern Africa region [9–11], threats to diagnostics posed by parasite *pfhrp2/3* gene deletions (which can cause false negative diagnostic test results) [12, 13], resistance of malaria vector mosquitoes to public health insecticides [14, 15], and the invasive vector species, *Anopheles stephensi* in the Horn of Africa [16–18]. All these factors threaten to undermine malaria control efforts in ways that are not sufficiently understood.

The WHO is tracking biological threats using the WHO threats map [19]. For *pfhrp2/3* gene deletions, there are already new tests, albeit more expensive, which are prequalified by the WHO that can detect these parasites [20]. Increased investments to improve surveillance of gene deletions is needed and investments in new diagnostics is essential and a cause for optimism. Insecticide resistance remains a significant challenge to be addressed decisively—PBO nets are now recommended, and other new generation nets are being evaluated [21]. The WHO recognizes *A. stephensi* as an efficient malaria vector in urban settings [22], and affected countries and their neighbours should urgently enhance surveillance and deploy novel tools. Given these threats, malaria stakeholders should be open to examining other potentially-transformative approaches such as genetically modified mosquitoes currently in early-stage development [23, 24].

Of particular concern is emerging signs of resistance to artemisinin, which is the backbone of current malaria treatment efforts in Africa [9–11]. Now confirmed in Uganda [9] and Rwanda [10, 11], artemisinin resistance, more accurately described as delayed parasite clearance, is emerging de novo in Africa and does not appear to be linked to the resistance in malaria parasites in south-east Asia, where this problem was first described [25]. Setting up effective surveillance systems is, therefore, critical to closely track this threat in the region.

### Severe malaria and the last mile

Combatting severe malaria is paramount for averting malaria deaths and depends on systems that support prompt treatment, referral for severe disease, and a full course of treatment to clear infection. However, the most severe malaria cases and deaths are often concentrated in areas where health systems are weakest, where prevention practices are most inadequate, and care workers least trained. Effective community-based approaches, particularly training and appropriately compensating community health workers will be key to reaching the unreached and preventing severe disease.

“…This is a Catch 22… if we try to build our health systems to reach the people furthest out, and at the greatest risk, using our least trained, least supplied workers, the system is then going to have to deal with severe malaria because we weren’t able to prevent it in the first place...the question is, how do we take the community outreach, and community health workers on the periphery, and make sure they’re sufficient in scale, have the right skills, and that they are adequately supervised and supplied?” –Dr. Steketee

### Funding gap

A consistent feature of global malaria programmes is that less than half of the necessary annual budget is actually available. A total of $3.3 billion was invested in 2020, compared to target of $6.8 billion. Moreover, to reach global targets, investments will need to increase by more than three times by 2030 to 10.3 billion per year. The current system relies on just a small number of major funders and budget needs are unlikely to be met even if these few sources increase their contributions. Further, the relative investment of countries has not increased despite economic growth.

“When you think about what’s stalled, population growth has not stalled, and that will continue, what’s stalled is the money. We’ve been working on efficiencies but there are limits to what we can achieve with efficiency alone.” –Dr. Steketee

## The future of malaria control

The malaria situation cannot be effectively tackled using current practices, highlighting the need for a more transformational approach, tailored to different epidemiological contexts. A drastic change in mindset is needed around the disease and its complexities.

“It has not sunk in that we need to do something drastically different. It is a mindset problem, we need to show greater flexibility, and understand we are facing a very complex problem…malaria is a problem to be solved, not simply a task to be performed.” –Dr. Alonso

### Lessons learned from the COVID-19 pandemic

There are important opportunities to learn from the COVID-19 pandemic. The pandemic brought the global malaria community together in a way not previously seen, to ensure a buffer against service delivery disruptions.

“It was really heartening to see that when there’s an emergency, we can work effectively across stakeholders to mount an effective response. COVID19 responses have also demonstrated to Ministries of Health that data matters – high-quality real-time data matters.” –Dr. Gardy

The pandemic has also shown that molecular data can provide important information on current and evolving trends over time, and that mathematical models can be valuable for exploring different intervention scenarios, an approach that is now also being utilized in the WHO-backed High burden, High impact response [2]. Perhaps most promising has been lessons learned from the development of the COVID-19 vaccine.

“...We’ve seen that things like a massive investment in de-risking multiple aspects of the vaccine production pipeline meant that you could very quickly get new products authorized, under Emergency Use Authorizations, and then eventually under full approval for use. We also saw the culmination of decades of work on mRNA vaccines…it’s working better than what we could have imagined. To hear that there’s now an mRNA pipeline for malaria vaccines is very exciting.” –Dr. Gardy

Innovative financing mechanisms will be needed moving forward to ensure sufficient and sustained funding. Resource mobilization seen during the COVID-19 pandemic shows when can be done when a disease is viewed as a global threat.

“COVID-19 may provide an opportunity – when countries in the global north have felt threatened there’s no limit to the money they spend – building on this momentum is a great opportunity to put the health agenda up front. Strengthening health systems is a key issue in the fight against malaria, it may not be considered malaria money, but is key to getting the commodities out.” –Dr. Alonso

### RTS,S malaria vaccine

In 2021, the RTS,S malaria vaccine became the first to be approved for widespread use; and the only vaccine currently available for any human malaria parasites. The vaccine is now recommended for children living in areas with moderate to high *Plasmodium falciparum* transmission. In addition to the modest efficacy demonstrated in earlier clinical trials [26, 27] and results of a consensus modelling programme [28], data from a WHO-backed pilot study in three countries, Kenya, Ghana and Malawi, suggest that the vaccine is feasible to deliver, safe and has a significant public health impact [29]. When provided in the context of both the expanded programme of childhood immunizations and other malaria control efforts, the vaccine increases access to prevention for vulnerable children—for instance reaching two thirds of children not protected by insecticide-treated nets (ITNs)—and is cost effective in areas with moderate to high transmission. The vaccine programme has already reached more than 900,000 children in three countries and generated among the most robust evidence for a malaria control tool ever.

It will be critical to think comprehensively about malaria control, including the vaccine, to ensure context-appropriate packages of interventions.

“…Putting one tool against another is really unhelpful, it’s bad public health…We have an armamentarium, we have a set of tools, and we need to look at what’s best in a particular circumstance….” –Dr. Alonso

During the evaluation of RTS,S there was a strong partnership between African scientists, the WHO, and several other players working jointly. For example, the Phase III trials were done in 11 different sites across nine African countries [26, 27], and the mathematical modelling done to support final decision-making had been conducted jointly by four different research groups [28]. There is an important opportunity to leverage benefits of such united approaches to improve outcomes for other technologies and malaria control programmes.

“RTS,S forces the malaria community to work with other departments of the ministry of health that are the custodians of the delivery platforms, such as EPI. Therefore, an added benefit of RTS,S is that it will force the malaria community to come out from a siloed space.” –Dr. Alonso

Working across disease portfolios can also maximize efficiencies in health systems.

“The more we can figure out how to work together on delivery platforms, the more we can see benefits across the board and use the limited (funding) envelope more effectively.” –Dr. Gardy

### Toward a unified vision and country-led decision-making

Finally, the future of malaria control will require moving toward country-led, unified visions and funding strategies. This includes ensuring evidence-based decisions and centering affected countries in those decisions.

“If a country has the data to show an area would benefit from a fifth round of seasonal malaria chemoprevention, who is anyone on this planet to tell them no? We need to break those attitudes, the lack of empowerment to countries, that lack of evidence-based decision making – only then will we be able to make progress.” –Dr. Alonso

There must also be a more coordinated response from different partners working within countries and a united strategy. This includes movement toward a single national strategic plan, that is costed properly and against which the investments from inside and outside of the country are aligned to achieve the agreed programme goals.

“Let’s get everyone at the table with one single plan, costed, that we all help develop and that we can all invest in. This is truly a partnership.” –Dr. Steketee

## Conclusion

The global malaria community is at an inflection point; progress has levelled off and multiple threats confront countries already hardest hit by the disease. A shift in mindset is urgently needed with truly innovative and collaborative approaches to malaria control. Reflecting on the 2021 WMR and its implications for the future, there is a critical opportunity to take-up lessons learned from the COVID-19 pandemic, including what is possible when the world comes together towards a common goal. Cutting edge research and development, as was seen in recent vaccine development, and improved surveillance, can pave the way to more transformational approaches. Finally, and most importantly, the future of malaria control must be led by affected countries, with unified and coordinated support from donors and partners.

## Data Availability

Not applicable.
